# Unusual manifestation of *Helicobacter cinaedi* infection: a case report of intracranial subdural empyema and bacteremia

**DOI:** 10.1186/s12879-016-2129-3

**Published:** 2017-01-07

**Authors:** Toshimasa Hayashi, Junko Tomida, Yoshiaki Kawamura, Masakazu Yoshida, Ikuyo Yokozawa, Shingaku Kaneko

**Affiliations:** 1 Division of Infectious Diseases, Maebashi Red Cross Hospital, Asahicho 3-21-36, Maebashi, Gunma 371-0014 Japan; 2 Department of Microbiology, School of Pharmacy, Aichi Gakuin University, Nisshin, Japan; 3 Division of Clinical Laboratory, Maebashi Red Cross Hospital, Maebashi, Japan

**Keywords:** *Helicobacter cinaedi*, Intracranial subdural empyema, Antimicrobial susceptibility testing, Case report

## Abstract

**Background:**

There have been various reports concerning *Helicobacter cinaedi* infections. However, few reports have examined central nervous system infections.

**Case presentation:**

A 52-year-old man was transferred from the local hospital because of a persistent headache and suspected intracranial subdural empyema. Neurosurgical drainage was performed via burr holes. Gram staining and results from abscess cultures were negative. The blood culture yielded *H. cinaedi*. He was given an antibiotic regimen consisting of 2 g of ceftriaxone twice a day, but the size of the abscess was not reduced in size at all after 3 weeks of treatment. Neurosurgical drainage was performed again, and the antimicrobial regimen was switched to 2 g of meropenem 3 times a day. The size of the abscess was reduced after 2 weeks of the second drainage and antimicrobial drug change to meropenem. After 4 weeks treatment with meropenem, the patient was discharged, and his symptoms had completely resolved.

**Conclusions:**

*H. cinaedi* infection should be considered in the differential diagnosis of subdural empyema cases for which Gram staining and abscess culture results are negative. Meropenem can be a first-line drug of choice or an effective alternative treatment for *H. cinaedi* central nervous system infections.

## Background

The first report of a *Helicobacter cinaedi* infection involved a man with proctitis in 1984 [1]. Since then, various foci of *H. cinaedi* infection have been reported. However, few reports have examined central nervous system (CNS) infections, and the optimal therapy for CNS infection is unknown. Here, we report a case of intracranial subdural empyema and bacteremia due to *H. cinaedi*, in which the patient experienced treatment failure after a maximum dose of ceftriaxone. Finally, he was treated successfully with adequate drainage and meropenem.

## Case presentation

A 52-year-old man with a history of epilepsy and drug eruption due to an amoxicillin/clavulanate was transferred from the local hospital because of a persistent headache and a suspicion of chronic subdural hematoma. There were no other symptoms before the persistent headache occurred, and he could perform his work normally. He had no history of head trauma and no meningeal irritation symptoms, such as neck stiffness. He kept an outdoor dog for years, but he had no contact with other animals, such as rats, hamsters, dogs, cats, birds, or monkeys, during the past year.

**Fig. 1:**
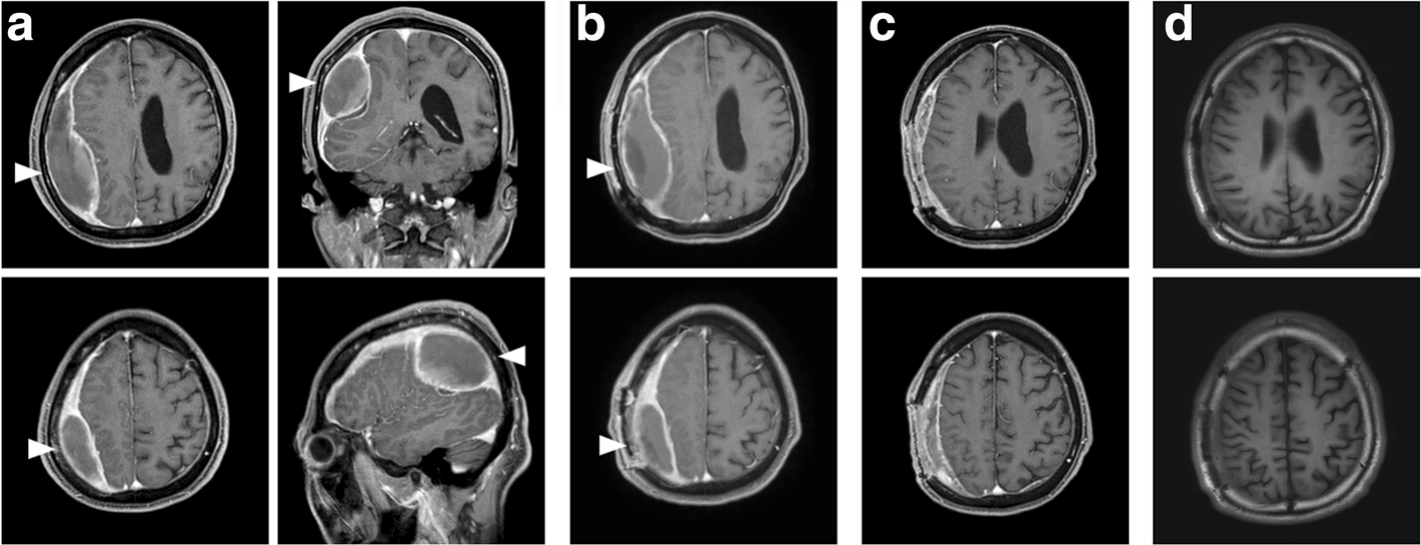
Nuclear magnetic resonance imaging of the head and brain. **a** Gadolinium-enhanced T1-weighted imaging (Gd T1WI) at the time of admission showed right subdural empyema (*white arrowhead*, 106 × 33 × 53 mm) with mixed low intensity. **b** Gd T1WI after 3 weeks of treatment. The size of the abscess was not reduced (*white arrowhead*, 109 × 35 × 60 mm). **c** Gadolinium-enhanced T1-weighted imaging 2 weeks after the second drainage and antimicrobial drug change to meropenem. The abscess was decreased in size. **d** T1-weighted imaging without contrast, 1 year after the treatment. There was no recurrence of the abscess

Computed tomography and nuclear magnetic resonance imaging of the head showed a right subdural mass with high and mixed density/intensity (Fig. 1a). Because of these findings, we suspected intracranial subdural empyema (SDE).

Neurosurgical drainage was performed via burr holes. Gram staining of the purulent material showed no bacteria, and he had no predisposition for sinusitis or periodontal disease. Therefore, we thought the possibility of anaerobic and aerobic gram-negative bacteria involvement was low. Two sets of blood cultures were drawn, and empirical antibiotic therapy was started with intravenous vancomycin targeting only aerobic streptococci and staphylococci. However, the results from abscess cultures were negative. After 7 days of incubation, the blood culture (BACTEC FX system, Nippon BD, Tokyo, Japan) yielded Gram-negative, long spiral-shaped bacillus. Microaerobic subculture on blood agar plates with hydrogen gas showed thin transparent colonies. Because of these findings, we suspected *Helicobacter cinaedi* intracranial subdural empyema. The human immunodeficiency virus antibody screening test administered just after surgery showed negative results. An additional two sets of blood culture specimens were drawn, and he was given an antibiotic regimen consisting of 2 g of ceftriaxone twice a day. The blood culture results were negative, but the size of the abscess was not reduced in size at all after 3 weeks of treatment (Fig. 1b).

**Fig. 2:**
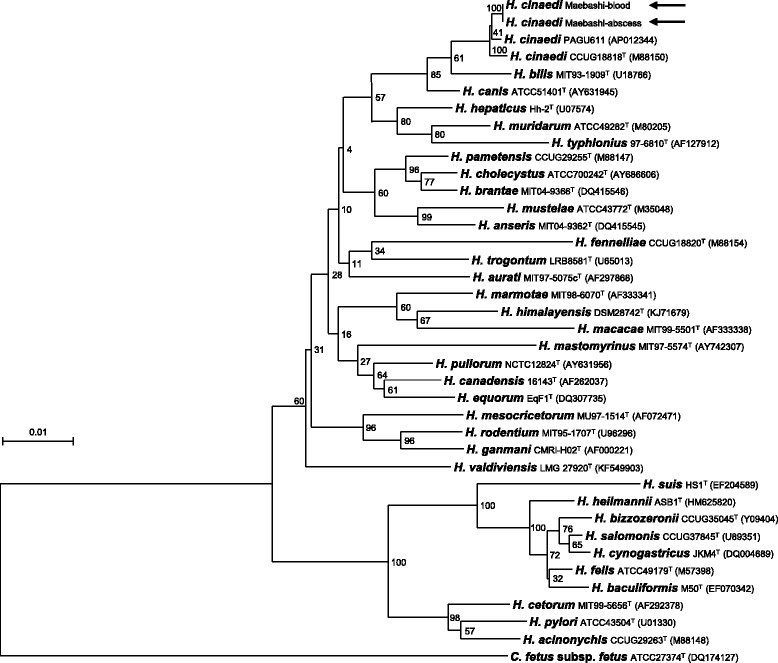
Phylogenetic tree of *helicobacter cinaedi*. Neighbor-joining tree showing the position within the species of the genus *Helicobacter*, based on 16S rRNA gene sequence. The numbers at the branching points are bootstrap values. *Campylobacter fetus* subsp. *fetus* was used as the out group. The numbers in parentheses are the accession numbers of the gene sequences. *Arrows* indicate the position of strains from the abscess or blood source in this case

Neurosurgical drainage was performed again, and the antimicrobial regimen was switched to 2 g of meropenem 3 times a day. Although the microaerobic culture of the abscess was negative, *H. cinaedi* was identified from the abscess and blood culture by *cdtB* virulence factor gene-based PCR. The 16S rRNA sequence analysis revealed with 100% similarity between the abscess and blood culture (Fig. 2). Antimicrobial susceptibility testing for *H. cinaedi* using the broth microdilution method revealed minimum inhibitory concentrations (MICs) of 4 μg/mL for ceftriaxone and 0.06 μg/mL for meropenem (Table 1). The size of the abscess was reduced after 2 weeks of the second drainage and antimicrobial drug change to meropenem (Fig. 1c). After 4 weeks treatment with meropenem, the patient was discharged, and his symptoms had completely resolved. The patient has continued visiting the hospital for more than 1 year after discharge, and there has been no recurrence to date (Fig. 1d).

**Table 1:** Antimicrobial susceptibility testing results of the *H. cinaedi* isolate

	MIC (μg/mL)
Penicillins	
Ampicillin	4
Amoxicillin	4
Carbenicillin	8
Piperacillin	4
Piperacillin/Tazobactam	4
Cephalosporins	
Cefepime	4
Ceftriaxone	4
Carbapenems	
Imipenem	0.06
Meropenem	0.06
Aminoglycosides	
Gentamicin	0.25
Kanamycin	0.5
Tetracycline	
Tetracycline	0.06
Macrolides	
Erythromycin	>64
Quinolones	
Ciprofloxacin	16
Levofloxacin	4
Moxifloxacin	0.5
Metronidazole	
Metronidazole	>64

## Conclusion

This case revealed two important clinical issues. First, *H. cinaedi* can cause an intracranial SDE. Secondly, treatment failure with ceftriaxone can occur when the MIC value for ceftriaxone is 4 μg/mL or higher in an *H. cinaedi* intracranial SDE case.

To our knowledge, this is the first published case of *H. cinaedi* intracranial SDE. There have been four reported cases of *H. cinaedi* central nervous system infections. Three cases concerned meningitis in adults [2–4], and the final case was a case of meningitis and bacteremia in a neonate [5]. None of those reported cases were associated with abscess formation. We must consider *H. cinaedi* as a causative organism of culture-negative intracranial SDE.

Treatment failure with ceftriaxone in cases of *H. cinaedi* intracranial SDE can occur if the MIC value for ceftriaxone is 4 μg/mL or higher. We initially chose ceftriaxone as definitive therapy because antibiotic regimens including ceftriaxone effectively treated *H. cinaedi* meningitis in the previous four case reports. Antimicrobial susceptibility testing (AST) for *H. cinaedi* isolates was not performed in these cases, probably because AST for *H. cinaedi* is too cumbersome to perform routinely in hospital laboratories [6].

However, in the present case, AST yielded useful information for changing the therapeutic strategy. With the doses normally used to treat bacterial meningitis, the concentrations of ceftriaxone in cerebrospinal fluid range from 2 to 8 μg/mL, and levels are nearly constant in children and adults [7, 8]. These concentrations are close to the MIC observed in this case (4 μg/mL), which may negate the culture results, but there is a possibility of treatment failure especially in the presence of an abscess. On the other hand, when 2 g of meropenem was administered every 8 h, the concentration in the cerebrospinal fluid was reported to be 1.29 μg/mL, even in the trough value [9], which is considerably higher than the MIC in this case (0.06 μg/mL). Therefore, meropenem can be a first-line drug of choice or an effective alternative treatment for CNS infection, especially when the MIC value is 0.06 μg/mL or lower.

In conclusion, *H. cinaedi* infection should be considered in the differential diagnosis of SDE cases for which Gram staining and abscess culture results are negative. AST for *H. cinaedi* isolates must be performed for cases of CNS infections. When the MIC value to ceftriaxone is 4 μg/mL or higher, treatment failure can occur. Meropenem can be a first-line drug of choice or an effective alternative treatment for *H. cinaedi* CNS infections.

## Abbreviations

AST: Antimicrobial susceptibility testing
CNS: Central nervous system
MIC: Minimum inhibitory concentration
SDE: Subdural empyema
